# Obsessive-Compulsive Disorder Associated with Posterior Cranial Fossa Meningioma

**DOI:** 10.1155/2017/8164537

**Published:** 2017-06-01

**Authors:** Nobuyuki Takeuchi, Etushi Kato, Kousuke Kanemoto

**Affiliations:** Neuropsychiatric Department, Aichi Medical University, Nagakute 480-1195, Japan

## Abstract

We report here a patient in whom the effects of a cerebellum mass may have led to development of obsessive-compulsive disorder (OCD). A 33-year-old woman showed symptoms of OCD, including obsessive worry about infection from tainted blood and repetitive confirmation, which worsened during pregnancy. She had comprehension in regard to her illness and no evidence of cognitive dysfunction and did not meet other DSM-5 criteria such as depression. One month after giving childbirth, the symptoms worsened, while headache and dizziness also developed. The Yale-Brown Obsessive-Compulsive Scale (Y-BOCS) score was 34. The patient was examined for a headache and a posterior cranial fossa meningioma was found. Following resection of the meningioma, the OCD symptoms were remarkably reduced (Y-BOCS score 10). There is only one previous report of pure OCD associated with a cerebellar mass and the present findings should help to elucidate the mechanism.

## 1. Introduction

Obsessive-compulsive disorder (OCD) is a psychological disorder characterized by obsessional ideas and compulsivity, such as repeatedly and irresistibly performing the same tasks [1]. Although the pathogenesis of OCD has not been clarified, neuroimaging findings have suggested that cortico-striato-thalamic circuits play an important role [2, 3], with many case reports also supporting that notion [4]. Details of affected patients are interesting and important to elucidate the etiology. Notably, there is only one previous case report of a cerebellum mass in a patient with OCD symptoms, which complicated automatic motor activity leading to dysphasia [5]. Here, we report a patient who developed OCD symptoms in conjunction with a posterior cranial fossa meningioma, who was successfully treated by resection of the mass. The patient provided consent for publication of the details of this case.

## 2. Case

### 2.1. Background History

The patient was right-handed and an only child. She was well adjusted and had no family history of note and also performed well in school. After graduating from university, she went on to graduate school and worked at a medical research institute until the age of 26 years, though her boss was strict and the work was difficult. At the age of 27 years, she began to experience appetite loss and a frequent stomachache and took a leave of absence from work for 6 months, after which she returned and felt well adjusted. She married at the age 30 years.

### 2.2. Clinical History

The patient was 33 years old when she first visited our clinic. At the age of 31 years, she became pregnant for the first time and started experiencing obsessively anxious feelings and concerns about an infection related to pregnancy, such as toxoplasma or mercury poisoning, and especially infection from tainted blood. After giving birth, the symptoms continued. Despite being able to perform housework and her job, she was unable to stop the obsessive thinking. For example, she would only use a self-service cash register because of worry that the shop clerk's hand might have been injured and soiled the products being purchased. Furthermore, she could not ride public transportation because of a strong anxiety that a passenger would cough blood or the seat would be contaminated.

The patient became pregnant again at the age of 33 years, at which time her feelings of anxiety widened to also focus on small stains. She would feel panic when touching something red, because she thought it might be blood and would also obsessively confirm whether something was clean or not for a long duration such as 15–20 minutes each time. She discarded more than 100 toothbrushes because the brush head had touched packing materials and was unable to use a blade, such as scissors or a razor. Even though the patient considered that these obsessive worries were nonsensical and abnormal, she was unable to control herself to reduce the anxiety or stop checking for blood. Finally, the symptoms led her to take a leave of absence from work.

She came to the Psychiatry Outpatient Department accompanied by her family and was given a clinical diagnosis of OCD. Cognitive function and mood were normal. Since the patient was pregnant, she rejected medication and only agreed to counseling. Full blood count, liver, kidney, and thyroid function results were normal. Approximately 1 month later, after returning to her parent's home to prepare to give birth, she was introduced to our hospital. The symptoms were continuing and we also gave a diagnosis of OCD, as she had a Yale-Brown Obsessive-Compulsive Scale (Y-BOCS) score of 34. The Structured Clinical Interview for DSM-5 (SCID-5) did not reveal any additional psychiatric comorbidities. Insight of her illness and cognitive function were preserved. There were no neurological symptoms such as abnormal motor activity, epilepsy, or tics, nor cerebellum-related symptoms. When hospitalized to give birth, the patient had excessive concern about possible infection and constantly checked for small stains. When some kind of stain was discovered, she called the nurse, which occurred more than 10 times each day. She rejected medication and could not receive counseling because the period of hospitalization was short.

**Figure 1:**
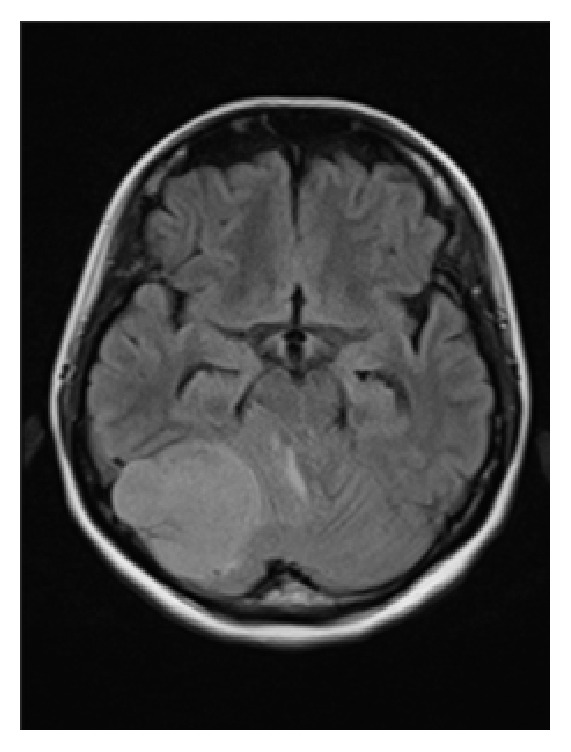
FLAIR MRI of the brain. Compression by the mass toward the brainstem caused deformation.

**Figure 2:**
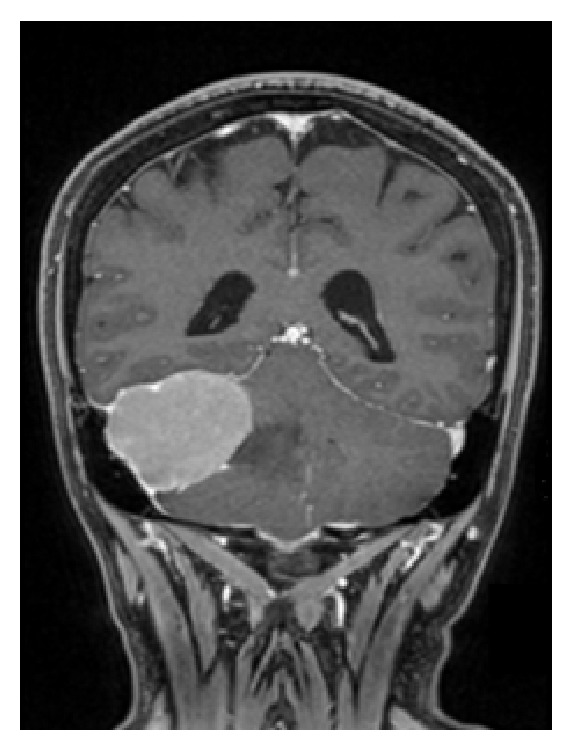
Coronal view of contrast-enhanced MRI scan showing a well-defined tumor with a clear margin. Compression by the mass toward the fourth ventricle caused deformation.

One month after childbirth, the symptoms worsened, while headache and dizziness also developed. T1, T2, FLAIR, and MRA brain magnetic resonance imaging showed a brain tumor in the right cerebellum measuring 4 cm in diameter, which was well defined and showed a clear boundary. As a result, the right cerebellum was compacted and edematous (Figures 1 and 2), while the right vertebral artery and fourth ventricle were also compressed. No other abnormalities were found in the brain.

**Figure 3:**
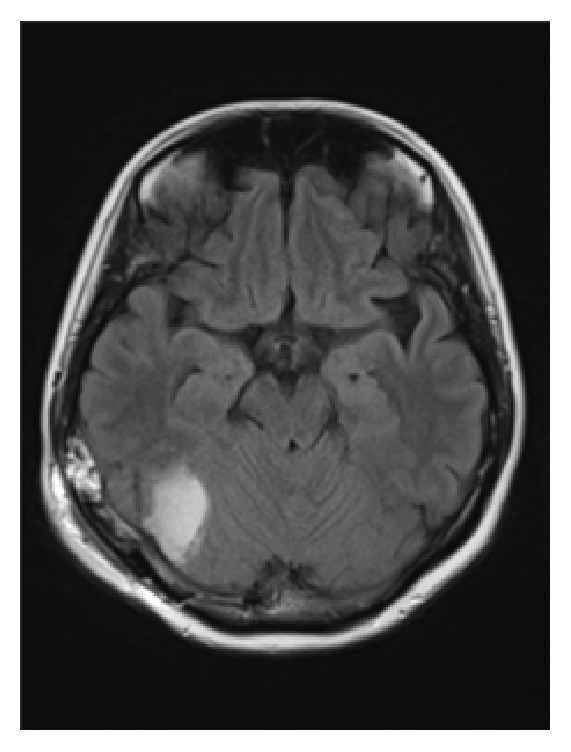
FLAIR MRI of the brain following resection. The meningioma was clearly resected, which resolved brain compression.

**Figure 4:**
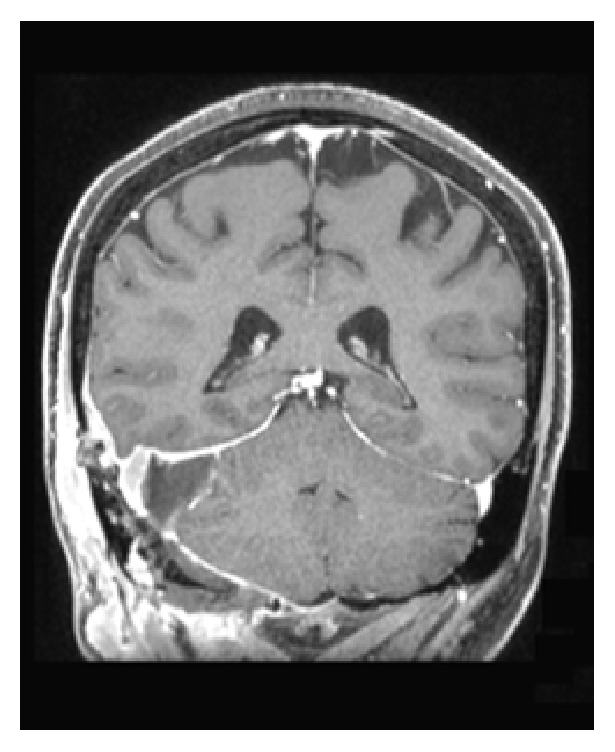
Coronal view of contrast-enhanced MRI scan following resection.

The patient underwent a procedure to resect the mass and the pathological findings revealed a meningothelial meningioma, WHO grade 1, while the diagnosis was posterior cranial fossa meningioma. Surgery to remove the meningioma was successful and compression was relieved, with only slight edema remaining around the contact parts of the tumor (Figures 3 and 4). Following the procedure, the intensity of anxiety and frequency of obsessive worry were remarkably reduced without medication or counseling. One month after undergoing resection, the patient was able to ride public transportation, use blades, and eat in public areas. With those subsequent improvements, she noted that should could not understand why she previously felt obsessively anxious and panicked (Y-BOCS score 12). Three months after the resection procedure, her obsessive anxiety regarding infection and performance of compulsive behavior such as confirming small stains were well controlled (Y-BOCS score 10). She no longer met the DSM-5 criteria of OCD as well as other psychiatric comorbidities.

## 3. Discussion

This is a case report of purely OCD symptoms that developed in association with a posterior cranial fossa meningioma. In the present patient, the OCD symptoms were considered likely to be associated with the presence of a cerebellum mass, because those rapidly improved following its resection without medication. There are many studies of OCD associated with brain lesions [4]. In reports of 3 different patients with OCD symptoms and a cerebellum lesion, 2 were complicated with automatic motor activity and dysphasia [5, 6], while there is only 1 known previous patient with purely OCD symptoms complicated with a cerebellum lesion [7]. That latter report is similar to the present in regard to both tumor location and clinical symptoms. After reviewing those studies, we considered the possible causes of OCD symptoms in our patient.

The first possibility is that the condition was caused by the mass compressing the basal ganglia. Theoretical models suggest that OCD is underpinned by functional and structural abnormalities of the corticostriatal circuits [2, 3]. In the present patient, compression from the meningioma extended to and around the basal ganglia as well as the cerebellum and deformed the fourth ventricle. It may be likely that the effect of the mass on the basal ganglia led to the OCD symptoms. Development of a meningioma during pregnancy has been reported, as up to 80% of benign meningiomas express the progesterone receptor [8]. In our case, OCD symptoms worsened with pregnancy and did not change during the postnatal period. The clinical history suggests that the meningioma became large during the first pregnancy, exacerbating her OCD symptoms, because of the effect by the mass on the basal ganglia. After delivery of her first child, the OCD symptoms remained unchanged because the tumor continued to slowly grow. During the second pregnancy, the tumor become larger and the OCD symptoms again worsened.

Another possibility is OCD caused by a disturbance of cerebellum functions. Although not related to OCD, psychotic symptoms in association with a cerebellum tumor have been reported [9–11]. In addition, Schmahmann el at. noted a relationship between the cerebellum and various psychiatric disorders including obsessive-compulsive symptoms [12]. The other OCD patient complicated with an arachnoid cyst who developed symptoms similar to those in our patient did not have compression, except for the area of the cerebellum. These findings support the notion that OCD symptoms are affected by a cerebellum lesion.

Nevertheless, it is important to consider the association between pathology and OCD symptoms. A meningioma is a commonly encountered type of benign intracranial mass, accounting for 13–26% of all primary intracranial tumors [13]. Although most affected patients are not complicated with symptoms, a retrospective study of 72 meningioma cases showed that 21% had only psychiatric symptoms, with affective disorders, a common presentation [14]. The relationship of a meningioma with OCD remains unclear.

Finally, there are some limitations to this study. We did not perform brain imaging before the patient gave birth or examination of tumor expression of the hormone receptor; thus there might not have been a causal relationship between the posterior cranial fossa meningioma and OCD symptoms in our patient. Furthermore, we cannot exclude the effects of pregnancy, because the complication of OCD is not rare in pregnant women, with a reported prevalence rate of 3.5% [15]. The cause in this case remains a matter of speculation and accumulation of additional cases is needed to elucidate OCD symptoms associated with a cerebellum lesion.
